# 3D-Printed Organ-Realistic Phantoms to Verify Quantitative SPECT/CT Accuracy for 177Lu-PSMA-617 Treatment Planning

**DOI:** 10.3390/ph18040550

**Published:** 2025-04-08

**Authors:** Lydia J. Wilson, Sara Belko, Eric Gingold, Shuying Wan, Rachel Monane, Robert Pugliese, Firas Mourtada

**Affiliations:** 1 Department of Radiation Oncology, Thomas Jefferson University Hospital, Philadelphia, PA 19107, USA; shuying.wan@ynhh.org (S.W.); firas.mourtada@jefferson.edu (F.M.); 2 Health Design Lab, Thomas Jefferson University, Philadelphia, PA 19107, USA; sara.belko@students.jefferson.edu (S.B.); rachel.monane@students.jefferson.edu (R.M.); robert.pugliese@jefferson.edu (R.P.); 3 Sidney Kimmel Medical College, Thomas Jefferson University, Philadelphia, PA 19107, USA; 4 Department of Radiology, Thomas Jefferson University Hospital, Philadelphia, PA 19107, USA; eric.gingold@jefferson.edu

**Keywords:** radiopharmaceutical therapy, SPECT imaging, 3D printing, ^177^Lu, partial volume effect, quantitative imaging

## Abstract

**Background/Objectives:** Accurate patient-specific dosimetry is essential for optimizing radiopharmaceutical therapy (RPT), but current tools lack validation in clinically realistic conditions. This work aimed to develop a workflow for designing and fabricating patient-derived, organ-realistic RPT phantoms and evaluate their feasibility for commissioning patient-specific RPT radioactivity quantification. **Methods:** We used computed tomographic (CT) and magnetic resonance (MR) imaging of representative patients, computer-aided design, and in-house 3D printing technology to design and fabricate anthropomorphic kidney and parotid phantoms with realistic organ spacing, anatomically correct orientation, and surrounding tissue heterogeneities. We evaluated the fabrication process via geometric verification (i.e., volume comparisons) and leak testing (i.e., dye penetration tests). Clinical feasibility testing involved injecting known radioactivities of ^177^Lu-PSMA-617 into the parotid and kidney cortex phantom chambers and acquiring SPECT/CT images. MIM SurePlan MRT SPECTRA Quant software (v7.1.2) reconstructed the acquired SPECT projections into a quantitative SPECT image and we evaluated the accuracy by region-based comparison to the known injected radioactivities and determined recovery coefficients for each organ phantom. **Results:** Phantom fabrication costs totaled < USD 250 and required <84 h. Geometric verification showed a slight systematic expansion (<10%) from the representative patient anatomy and leak testing confirmed watertightness of fillable chambers. Quantitative SPECT imaging systematically underestimated the injected radioactivity (mean error: −17.0 MBq; −13.2%) with recovery coefficients ranging from 0.82 to 0.93 that were negatively correlated with the surface-area-to-volume ratio. **Conclusions:** Patient-derived, 3D-printed fillable phantoms are a feasible, cost-effective tool to support commissioning and quality assurance for patient-specific RPT dosimetry. The results of this work will support other centers and clinics implementing patient-specific RPT dosimetry by providing the tools needed to comprehensively evaluate accuracy in clinically relevant geometries. Looking forward, widespread accurate patient-specific RPT dosimetry will improve our understanding of RPT dose response and enable personalized RPT dosing to optimize patient outcomes.

## 1. Introduction

The last decade has seen several targeted radiopharmaceutical therapies (RPTs) proven safe and effective, with more promising agents in development [1]. However, further optimization of this targeted therapy will necessitate patient-centric dose-response evaluation to enable tailoring RPT delivery based on tumor response and active monitoring of toxicity [2]. Patient-specific dosimetry has been shown to be predictive of RPT response for a few RPT agents [3,4,5]. For instance, a recent randomized, multicenter phase 2 trial (DOSISPHERE-01) evaluated personalized versus standard dosing of Y-90–microsphere therapy in patients with locally advanced hepatocellular carcinoma and demonstrated significant improvement in response for the personalized-dose arm [4].

Although notable, the DOSISPHERE-01 findings are not surprising as tissue response to patient-specific doses is the hallmark of radiation oncology both for external beam and brachytherapy modalities. In radiotherapy, the net uncertainty (k = 2) for an individual patient’s dose delivery is within 5% for external beam [6] and somewhat higher at 15–20% for brachytherapy [7]. However, for RPT the uncertainty of radiation dose delivery is much higher and can be over 100%, although the use of single-photon emission computed tomography (SPECT) can reduce it to 50–70% [8].

Even with SPECT-based dose estimation, several known factors contribute to RPT radiation dose uncertainty. For example, attenuation and scatter represent physical limitations that introduce uncertainty into the SPECT acquisition, and time-activity curve fitting and absorbed dose model assumptions introduce theoretical uncertainty, which together influence the net dosimetric uncertainty. Critically, uncertainties in the earlier steps of any RPT dose-estimation workflow can compound in later steps to exacerbate uncertainties. Therefore, one crucial step in RPT dose estimation is to accurately determine the total number of decays (i.e., radioactivity) per organ or voxel.

Recent advances in quantitative SPECT imaging show promise to allow meaningful absorbed dose estimation with reasonable total uncertainty [9]. Although novel SPECT reconstruction methods have improved the accuracy of the measured radioactivity on the voxel level, the poor spatial resolution of SPECT can lead to a phenomenon known as the partial-volume effect (PVE) [10]. The PVE leads to blurring of the true, heterogeneous radioactivity distribution and is a major source of uncertainty in SPECT-based RPT dose estimation [11].

Conventional methods to correct for PVEs use spherical or cylindrical phantoms of varying diameters to determine the algorithm for converting an uncertain imaged radioactivity into the true radioactivity within a volume of interest [12,13]. However, the limited anatomic relevance of spheres and cylinders poses substantial challenges for translating conventionally determined PVE corrections into patient-specific RPT dosimetry. Tran-Gia and Lassman investigated the clinical relevance of conventional PVE corrections by employing 3D printing to develop single- [14] and double-compartment [15] ellipsoid phantoms, meant to more closely represent kidney anatomy. The authors went on to compare the partial-volume recovery coefficients (RCs) determined from their two-compartment ellipsoid phantoms to those of conventional spherical and elliptical phantoms of equal volumes [15]. Although their study revealed little difference between the sphere- and ellipsoid-based RCs, the difference between RCs from spheres and two-compartment ellipsoid phantoms was 31.7%. More recently, Jessen et al. further advanced the possible clinical realism of RPT phantoms by developing a grid-based 3D printing method that could produce non-uniform radioactivity distributions [16] and tested their grid-based 3D printing method in kidney and thyroid phantoms based on digital phantom anatomy. More recent studies have investigated incorporating additional clinical realism by basing 3D-printed, organ-realistic phantoms off real patient anatomy [17,18]. However, no prior studies to our knowledge have created patient-derived RPT phantoms that could holistically represent the relative separation and orientation of left- and right-sided structures as well as surrounding tissue heterogeneities, all of which may affect SPECT accuracy via contributions of scatter and crosstalk. The goal of this study was to develop a workflow for designing and fabricating patient-derived organ-realistic phantoms with fillable compartments and evaluate a possible clinical application via end-to-end testing as part of the commissioning for patient-specific RPT radioactivity quantification.

## 2. Results

### 2.1. Phantom Design and Fabrication

Printing the kidney phantoms used 284 mL of FormLabs clear V4 resin with 162 mL for the positioning base, 60 mL for the left kidney, and 62 mL for the right kidney. The total material cost of the phantom system was USD 42, based on the value at the time of printing. Total print time on one printer for one phantom system (one positioning base, one left kidney, and one right kidney) was less than 23 h total, divided between approximately 9 h for the base and 7 h each for the left and right kidneys.

Printing the parotid-containing facial model used 381 mL of FormLabs clear V4 resin with 194 mL for the left side and 187 mL for the right side. The total material cost of the phantom system was USD 57, based on the value at the time of printing. Total print time on one printer for one phantom system (i.e., one left side and one right side) was less than 61 h, divided between approximately 28 h for the left side and 33 h for the right side.

The total cost for both phantoms was USD 248 including the cost of the material and one Form 3 Resin Tank (USD 149), but excluding the cost of the printer itself. The total print time for both phantoms was less than 84 h.

### 2.2. Geometric Verification

Table 1 shows the measured volumes of the organ models at three timepoints in the phantom design and fabrication workflow as well as the relative difference between the final printed volume and the volume of the final computer-aided design (CAD) model sent for printing. The data revealed that the final printed chambers were uniformly larger than the original representative patient anatomy, with an average volume error of 3.86 cc (2.0%) between the original contour and the final CAD model for the four kidney and parotid full chambers, and another average volume error of 3.43 cc (4.5%) between the final CAD model and the image of the printed phantom for all six chambers.

### 2.3. Leak Testing

**Figure 1:**
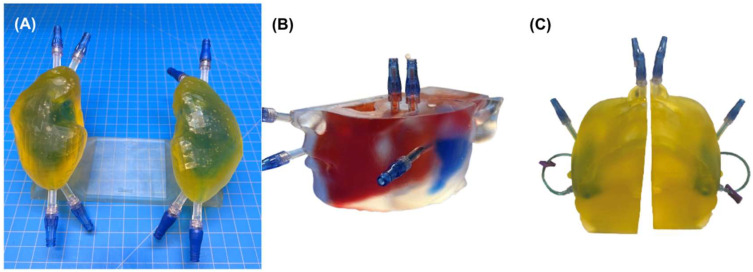
Pictures of the finished kidney and parotid phantoms following leak evaluations via dye penetration testing. (**A**) Kidney dye penetration test with blue-dyed water in the medulla chambers and yellow-dyed water in the cortex chambers. (**B**) First-phase parotid phantom leak testing with red-dyed water in the bone chamber and blue-dyed water in the parotid chamber. (**C**) Second-phase parotid phantom leak testing with yellow-dyed water in the soft-tissue chambers and blue-dyed water in the parotid chambers.

Visual inspection after 24 h revealed no evidence of color mixing among independent chambers in kidney or parotid phantoms. Figure 1 shows images of the phantoms post-leak-testing, depicting clear separations between differently dyed water. The absence of color mixing confirmed a successful dye-penetration test, supporting watertightness of the various phantom chambers.

### 2.4. Clinical Feasibility Testing

**Figure 2:**
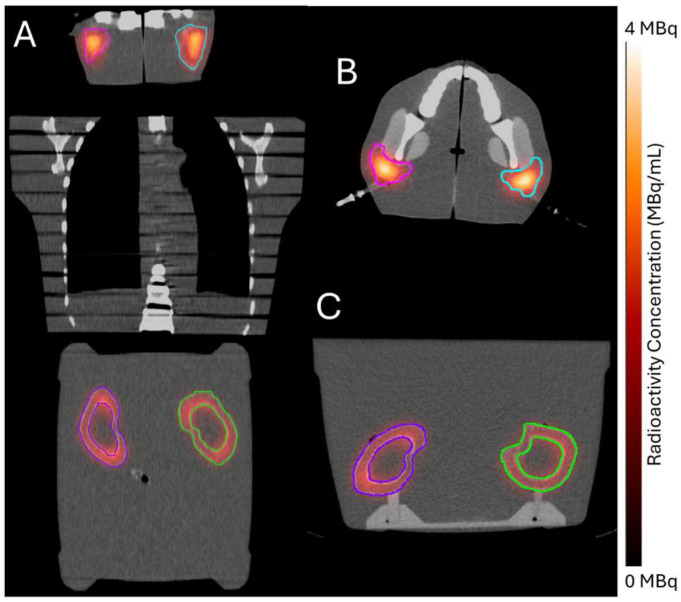
Representative views of the quantitative single-photon emission computed tomography (SPECT) image (color wash) overlaid on the computed tomography (CT) image (grayscale) collected 96 h post injection. (**A**) Coronal view of full image acquisition. (**B**) Axial view of representative slice through the parotid chambers. (**C**) Axial view of representative slice through the kidney phantom. Colored contours indicate the left parotid (cyan), right parotid (magenta), left kidney cortex (green), and right kidney cortex (purple).

End-to-end testing of the 3D-printed phantoms successfully enabled injection, imaging, and decay-to-background (73 days post injection) of ^177^Lu-PSMA-617 without evidence of leakage or radioactive contamination. Figure 2 shows representative slices of the SPECT/CT image collected at 96 h post injection. Visible radioactivity outside of the contoured chamber boundaries was attributed to partial volume effects, which result from the limited spatial resolution of SPECT systems [10].

#### Radioactivity Verification

**Figure 3:**
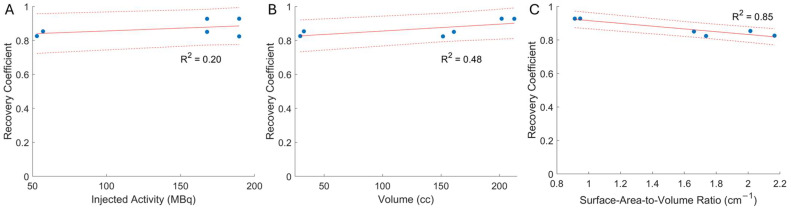
Scatter plots illustrate the sensitivity of the recovery coefficient to the (**A**) injected radioactivity, (**B**) region-of-interest volume, and (**C**) ratio of the region-of-interest surface area to its volume. Red solid lines show the fit line determined by standard linear least-squares analysis and characterized by the r-squared value displayed on each plot.

Table 2 shows the results of the radioactivity verification measurements, revealing that the quantitative image systematically underestimated the known injected radioactivity, as is characteristic of the spill-out partial volume effect. The mean error between imaged and decay-corrected injected activities was −17.0 MBq (−13.2%). Figure 3 depicts scatter plots of the sensitivity of the recovery coefficient to the (A) injected radioactivity, (B) region-of-interest volume, and (C) ratio of the region-of-interest surface area to its volume. Red solid lines show the fit line determined by standard linear least-squares analysis and characterized by the r-squared value displayed on each plot. The recovery coefficient showed a weak positive correlation with injected radioactivity and chamber volume, and a strong negative correlation with the surface-area-to-volume ratio.

## 3. Discussion

This work represents the first development, to our knowledge, of patient-derived 3D-printed phantoms for kidney and parotid glands, inclusive of anatomically accurate relative orientation of left- and right-sided structures and surrounding tissue heterogeneities. We additionally performed an end-to-end test of the intended clinical application for our organ-realistic phantoms to assess the accuracy of quantitative ^177^Lu-PSMA-617 SPECT imaging. Quantitative SPECT imaging is an essential component of commissioning a clinical patient-specific RPT dosimetry platform and professional standards recommend annual re-testing to verify system stability [19]. Our findings revealed that it is feasible to economically and efficiently generate phantoms in-house that (1) faithfully represent organic curvatures, shapes, orientations, and heterogeneities of patient anatomy, (2) are watertight to contain liquid radionuclide injection, and (3) evaluate and characterize the accuracy of quantitative SPECT image reconstruction for the purposes of patient-specific RPT dosimetry. Our findings are important as RPT soars in popularity [1] and commercial tools increase the accessibility of routine, patient-specific dosimetry [2].

Although our work is the first to evaluate patient-derived RPT phantoms, our results are largely consistent with those in the literature. Others have noted the cost-effectiveness of 3D printing for generating RPT phantoms [14,16,20]. Total resin material used, printing time, and monetary cost were comparable between our kidney phantoms and that reported by Jessen et al., in consideration of design differences (e.g., their grid-based printing technique, our positioning base) [16]. Differences between the radioactivity magnitude, target-to-background ratio, and kidney sizes make it difficult to directly compare our recovery coefficient results to others’. However, despite the differing experimental approaches, Grings et al. [18] and Salvadori et al. [17] both reported a strong, negative correlation between the surface-area-to-volume ratio and recovery coefficient, as we found here (Figure 3). Our kidney phantoms were characterized by smaller surface-to-volume ratios (i.e., 0.9 cm^−1^ for whole kidney; 1.7 cm^−1^ for kidney cortex) than the other two studies (i.e., 1.4–2.8 cm^−1^ for whole kidney [17,18]; 2.4–3.9 cm^−1^ for kidney cortex [18]) and, accordingly, our recovery coefficients were somewhat larger (i.e., 0.8 for our study, 0.6 for Tran-Gia et al. [15], and 0.5 for Grings et al. [18]). Such differences are not surprising since other critical factors influence the recovery coefficients. For instance, the MIM SurePlan MRT planning system uses SPECTRA Quant software to obtain the quantitative SPECT reconstruction of the TEW projection data, which is different from the prior publications using the commercially available reconstruction methods xSPECT Quant or Flash3D (Siemens Healthineers). In fact, Tran-Gia et al. [15] reported differences between xSPECT and Flash3D.

Our study has several notable strengths. It is the first to report end-to-end testing of patient-derived 3D phantoms complete with relative organ orientations and surrounding heterogeneities. Also, our study is highly clinically relevant as it used commercially available, FDA-approved quantitative reconstruction software. What is more, our leak-test procedure will be indispensable to the clinics implementing and maintaining patient-specific RPT dosimetry workflows as it demonstrated a critical step for achieving radiation safety and experimental success in the required commissioning and routine quality-assurance procedures. Finally, our 3D-printed model was based on a parametric approach, making it easily scalable to other patient sizes or shapes to facilitate the future work that we have planned and potential collaboration with other groups and centers.

Our study also had some limitations. PVEs are known to be sensitive to many experimental parameters including region-of-interest volume, radioactivity concentration, and target-to-background ratio. In this work, we only generated one set of kidney phantoms and one set of parotid phantoms; however, this was not considered a serious limitation because between the kidneys and parotids, the phantoms included a range of morphologies, volumes, and radioactivity concentrations. Furthermore, our method of applying a parametric approach will support our future work to investigate PVEs in other patient-derived anatomy, radioactivity concentrations, and target-to-background ratios. Additionally, we only considered a homogeneous radioactivity distribution within our kidney and parotid chambers. Although a homogeneous distribution is clinically representative of parotid RPT uptake [21], the kidneys are known to exhibit inhomogeneous uptake distributions [22]. However, the most marked inhomogeneity is between the kidney cortex and medulla, which was represented in our two-compartment kidney phantom, and future work can implement existing methods to induce inhomogeneous distributions within the cortex chamber [16]. Finally, we only considered region-based PVE correction techniques. Future work is needed to investigate and optimize voxel-based PVE correction methods [17].

In terms of the 3D printing errors (shown in Table 1), it has been shown that FormLabs clear stereolithography (SLA) printing for single, less complex organ models has an average relative printing error (mm) of 1.52% (1.28% SD) without accounting for CAD post-processing to improve printability [23]. As the model’s complexity (e.g., multi-chamber models) and the need for post-processing to improve printability increases, the chances of printing errors may increase. Future work is needed to investigate and optimize CAD post-processing methods to minimize printing error (%).

The work presented here is a fundamental critical step to improve upon the accuracy of treatment planning dose predictions. Recently, Ells et al. reported a meta-analysis of absorbed dose of ^177^Lu-PSMA-617 and ^177^Lu-PSMA-I&T from twenty-nine published articles comprising 535 patients [24]. They concluded that the dosimetry methodologies were strikingly heterogeneous among studies, emphasizing the need for standardization. Interestingly, a few studies mentioned if the PVE correction method was applied to obtain the reported organ doses. The importance of our study of state-of-the-art 3D printing technology is to pave the way for standardization efforts for SPECT/CT quantifications for radiotheranostics dose reporting and the eventual treatment planning similar to the radiation oncology process.

## 4. Materials and Methods

### 4.1. Phantom Design and Fabrication

#### 4.1.1. Representative Anatomy

We designed and generated organ-realistic phantoms to represent the kidneys and parotid glands, as two organs considered to be at risk from RPT with ^177^Lu-PSMA-617 [25]. We modeled our organ-realistic phantoms off representative patient anatomy using medical images. RPT dose calculations are known to be sensitive to the size and shape of regions of interest, with limited physical realism contributing to either over or underestimation of the radiation absorbed dose [26]. The use of medical images allows for derived organ contours to provide boundary conditions that are closer to patient anatomy than what is represented by standard nuclear medicine reference phantoms. The consideration of more organic, realistic shapes and curvatures throughout the RPT dosimetry commissioning workflow is expected to yield more accurate recovery coefficients that are directly relevant to critical organ shapes and sizes to improve dose calculations for future treatment planning. Representative male kidney and parotid anatomies were selected from the TJU Radiation Oncology computed tomography (CT) and magnetic resonance imaging (MRI) databases, respectively, by identifying the images with organ-of-interest volumes approximating previously published population averages [27,28,29]. We selected anatomy representative of the population average to investigate this 3D printing method in clinically relevant anatomy. However, we stress that the workflow was specifically designed to be easily adapted to represent a wide range of organ sizes in future studies by the implementation of scalable parametric CAD models. Either computed tomography (CT) or MR image fidelity is acceptable for both kidney and parotid gland contour delineation for radiation oncology treatment planning. In general, thinner-sliced image sets (e.g., CT) produce smoother contours in segmentation and require less post-processing in computer-aided design programs. Board-certified subject-matter experts (i.e., an Ear Nose and Throat physician for the parotids and a Urologist for the kidneys) reviewed the selected images and confirmed their suitability. Commercial clinical software (MIM Version 7.1.6, MIM Software Inc., Beachwood, OH, USA) de-identified the selected images and exported them for segmentation.

#### 4.1.2. Segmentation and Computer Aided Design

**Figure 4:**
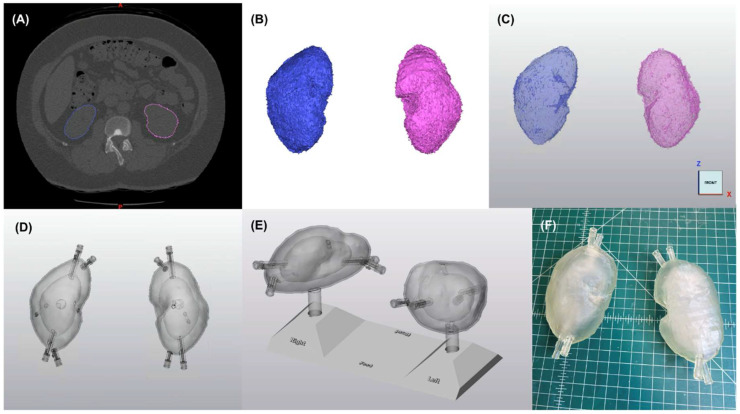
(**A**) Segmentation of nonpathological kidneys. Pink and blue lines outline the left and right kidneys, respectively. (**B**) Unprocessed left and right kidney three-dimensional (3D) parts created in Materialise Mimics. (**C**) Unprocessed 3D parts in Materialise 3-Matic. (**D**) Left and right kidney models following computer-aided design processing. (**E**) Left and right kidney models simulated in labeled base. Labels read Right, Left, Head, and Foot. (**F**) 3D-printed left and right kidneys with two chambers.

**Figure 5:**
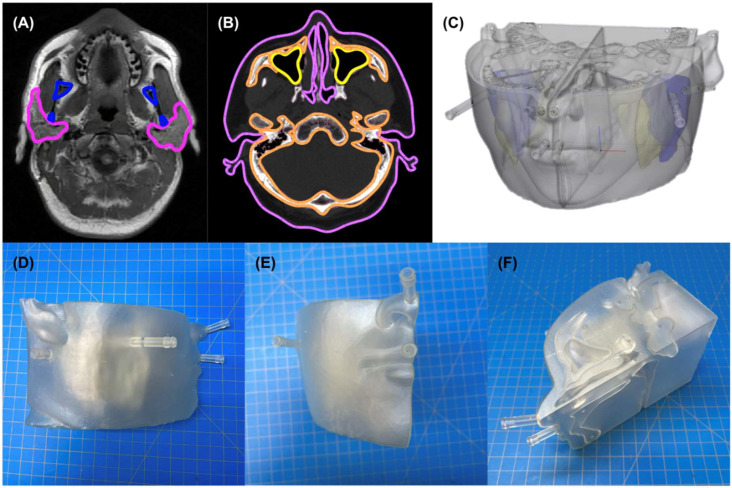
(**A**) Segmentation of nonpathological parotids (pink) and mandible (blue). (**B**) Segmentation of nonpathological sinuses (yellow), soft tissue (purple), and bone (orange). (**C**) Processed lower face computer-aided design model divided into two halves along the sagittal midline plane with masseter muscle (yellow) and chambers for separate bone, soft tissue, and parotid (blue) compartments; (**D**) side, (**E**) front, and (**F**) top-medial views of three-dimensional-printed right parotid model with three chambers for separate bone, soft-tissue, and parotid compartments. Note that only soft-tissue and parotid compartments had valves for on-demand filling.

Preset soft tissue thresholds and edit masks tools in image processing software (Materialise Mimics Version 25.0, Materialise NV, Leuven, Belgium) isolated bilateral kidney in the representative CT image, and parotid glands, esophagus, sinuses, masseter muscle, bone (i.e., mandible, maxilla, base of skull), and soft-tissue surface from the representative magnetic resonance (MR) image. The image processing software converted the resulting segmentations (Figure 4A and Figure 5A,B) into rough 3-dimensional (3D) parts (Figure 4B), which were transferred to CAD software (Materialise 3-Matic Version 17.0, Materialise NV, Leuven, Belgium) (Figure 4C) for further processing to improve 3D printability.

CAD modifications of the left and right kidneys (Figure 4D,E) included:

1. Wrapping, smoothing, and reduction in triangles on the surface of the kidney parts to produce a more natural appearance;
2. Hollowing to create a 1.5 mm thick wall, minimizing potential radiation attenuation from the 3D-printed material;
3. Creation of two chambers in each kidney by duplicating the hollow parts and reducing the organ size by 0.31 to represent (1) the cortical (outer) chamber, containing 67% of the model’s volume and (2) the medulla (inner) chamber, containing 31% of the model’s volume [30];
4. Adding angled support structures to secure the inner medulla chamber to the outer chamber wall;
5. Creation of two luer ports per chamber (i.e., four in total per kidney) to be used with standard one-way valves to inject water and/or ^177^Lu-PSMA-617 into each chamber;
6. Design of a peg and base system (180-degree lock-and-key design) to position kidneys in their anatomic orientations (i.e., distance between kidneys and distance above scanner bed) while imaged and immersed in water; and
7. Labeling the base with indicators of left, right, head, and feet, to ensure proper and consistent orientation.

CAD modifications of the parotids (Figure 5C) included:

1. Wrapping, smoothing, and reduction in triangles on the surface of the bone, soft tissue, and parotid parts to produce a more natural appearance;
2. Boolean subtraction of the sinuses and esophagus from the soft tissue (to simulate naturally occurring air pockets);
3. Mirroring of the left parotid across the midline to produce a matched right parotid structure;
4. Hollowing and creation of three chambers in each side of the head representing parotid, skull, and soft tissue, respectively, each with 1.5 mm walls;
5. Creation of two luer ports in each of the soft tissue and parotid chambers to be used with standard one-way valves to inject water and/or 177Lu-PSMA-617; and
6. Creation of simple access holes for injecting bone-mimicking material into the skull chamber.

We exported the finalized models as 3D files (surface tessellation language, STL) for 3D printing.

#### 4.1.3. 3D Printing

We printed the kidneys (Figure 4F) and their positioning base and parotid models (Figure 5D–F) using stereolithography (SLA) on a FormLabs 3B printer (www.formlabs.com, Somerville, MA, USA). SLA is an additive manufacturing type, also known as vat photopolymerization or resin 3D printing. Components include a build plate, photosensitive resin, a vat (or tank), and UV light (or laser). The printing process occurs as the build plate dips down into liquid resin and the UV laser passes underneath it, curing that layer. The build plate then lifts to allow the excess resin to drip back into the tank, before the build plate lowers again to add the next layer. The process repeats for hundreds of layers until the model is finished. We chose SLA over other 3D printing techniques because of its potential to print highly accurate, isotropic, watertight models, in myriad materials. For example, SLA could print the models in 25, 50, or 100 µm layers compared to the 100–300 µm layer thickness common of fused filament fabrication printing [31,32], allowing for better resolution of fine details via SLA. We printed the models in FormLabs clear resin because the semitransparency allowed for visual confirmation of interior structures and its cost-effectiveness at USD 149 per liter of material.

Formlabs’ PreForm print preparation software (version 3.30.0) prepared the STL files for 3D printing. We strategically arranged the right and left kidneys, positioning base, and parotid models on the build plate such that one port of each chamber acted as a drain hole allowing uncured resin to drain out of the model and back into the tank while printing. Left and right multi-chambered kidney, positioning base, and multi-chamber parotid models were printed as individual parts, each within a single print. Table 3 shows the print parameters used. Note that we printed the parotid phantoms at a smaller layer thickness than the kidney phantoms to more precisely represent the complex detail included in the partial-face anatomy of the parotid phantoms. We post-processed the models in an agitator containing isopropyl alcohol for 10 min followed by manual isopropyl-alcohol flushing of internal uncured resin until resolved. Finally, we cured the models in the Formlabs Form Cure for 15 min at 60 °C in accordance with the instructions published by the material manufacturer and manually removed any 3D-printed external support structures.

#### 4.1.4. Tissue Heterogeneity Representation

We represented tissue heterogeneities surrounding the parotid glands using readily accessible and easily integrated materials that reasonably mimicked the radiographic properties of the corresponding tissues. We filled the bone chamber with calcium chloride (CaCl_2_)–water solution (40% CaCl_2_ by weight) formulated to provide an average Hounsfield unit (HU) value of 944 to effectively mimic that of bone, which typically ranges from 500–1500 HU [33]. The masseter muscles were printed as solid structures to be represented by the 3D-printed material (FormLabs clear resin) as preliminary studies revealed it had an average HU value of 118, aligning with the −29 to 150 HU range of muscle tissue [34]. Water (0 HU) represented all soft tissues (i.e., kidneys, parotids, and surroundings aside from those listed above), as is standard for radiation imaging and dosimetry experiments. Sinus and esophagus cavities were hollow to ambient air fill.

#### 4.1.5. Phantom Watertight Sealing

After filling with bone-equivalent solution, we sealed the bone chamber using uncured resin material and spot laser welding. All other chambers were capped with generic one-way valves with luer lock ends. Two ports in each chamber (i.e., four ports per kidney (Figure 4F) and four ports for each half-lower-face segment (Figure 5D)) allowed for flexible experimental design, enabling nearly limitless combinations of radiopharmaceutical concentrations to be filled into the various soft-tissue compartments.

### 4.2. Geometric Verification

We verified the geometric fidelity of the printed models by comparing volume measurements at three timepoints in the workflow to design and fabricate the phantoms. First, we measured the volume of the original contours of the left and right kidneys and the left parotid from the representative patient anatomy (Materialise Mimics Version 26.0). Next, we measured the volumes of the finalized CAD models of the left and right kidney medullas and cortices and the left parotid (Materialise 3-Matic Version 19.0). Finally, we measured the left and right kidney medullas and cortices and the left and right parotids as visualized in a CT image of the full printed models (MIM Version 7.1.6). Considering the chamber volumes at three distinct timepoints enabled us to evaluate how accurately the printed model matched representative patient anatomy and consider to what extent discrepancies were attributable to CAD transfer and processing or to printing imprecision.

### 4.3. Leak Testing

We tested the watertightness of the phantoms, including the absence of possible leakage between the chambers intended to represent independent tissue compartments, via 24 h dye penetration testing [35]. The thickness of the parotid phantom hindered visualization of interior chambers and necessitated leak testing in two phases. The first-phase dye-penetration test involved red food dye added to the CaCl_2_–water solution in the bone chamber, blue-dyed water in the parotid chamber, and clear (undyed) water in the soft-tissue chamber. The second-phase test began after the full completion of phase one with yellow food dye added to the water in the soft-tissue chamber. Kidney phantoms received blue-dyed water to the medullar (i.e., inner) chambers and yellow-dyed water to the cortical (i.e., outer) chambers for a single dye-penetration test. Chamber filling leveraged the two-valve system of each chamber by using one valve as an air vent and the other to fill the cavity. We agitated the phantom and manipulated its orientation to facilitate the removal of air bubbles during the filling process.

We left the printed phantoms to stand undisturbed for 24 h before visually checking for color mixing between the various chambers, which would indicate leakage.

### 4.4. Clinical Feasibility Testing

We tested the suitability of the 3D-printed phantoms for their intended end use by completing a full end-to-end test.

#### 4.4.1. Radioactivity Injection

We injected known activities of ^177^Lu-PSMA-617 into the kidney cortex and parotid chambers of the 3D-printed phantoms. Prior to radioactivity injection, all chambers contained water. Radioactivity quantification and injection involved:

1. Removing 50 mL of water from each of the kidney cortex and parotid chambers to create space for ^177^Lu-PSMA-617;
2. Drawing ^177^Lu-PSMA-617 into a 20 mL syringe;
3. Measuring the radioactivity using a dose calibrator (Capintec CRC-15R) that had previously been cross-calibrated using a reference radioactivity of 177Lu-PSMA-617 following manufacturer guidelines;
4. Injecting the radioactivity into the partially emptied (from step 1) chamber;
5. Flushing the syringe with water at least twice to transfer residual radioactivity from the syringe and luer valve system into the chamber;
6. Measuring the residual radioactivity left in the syringe using the dose calibrator, and
7. Filling the chamber to capacity with water and removing any air bubbles.

We identified target radioactivity concentrations for each chamber based on prior publications [15] and our preliminary investigations, aiming to optimize count rates to keep deadtime losses low in the subsequent image acquisition. Table 4 shows the net injected radioactivity in each chamber. Other soft-tissue chambers were left filled with water (i.e., no radioactivity; region of interest to background ratio of infinity).

#### 4.4.2. SPECT/CT Image Acquisition

**Figure 6:**
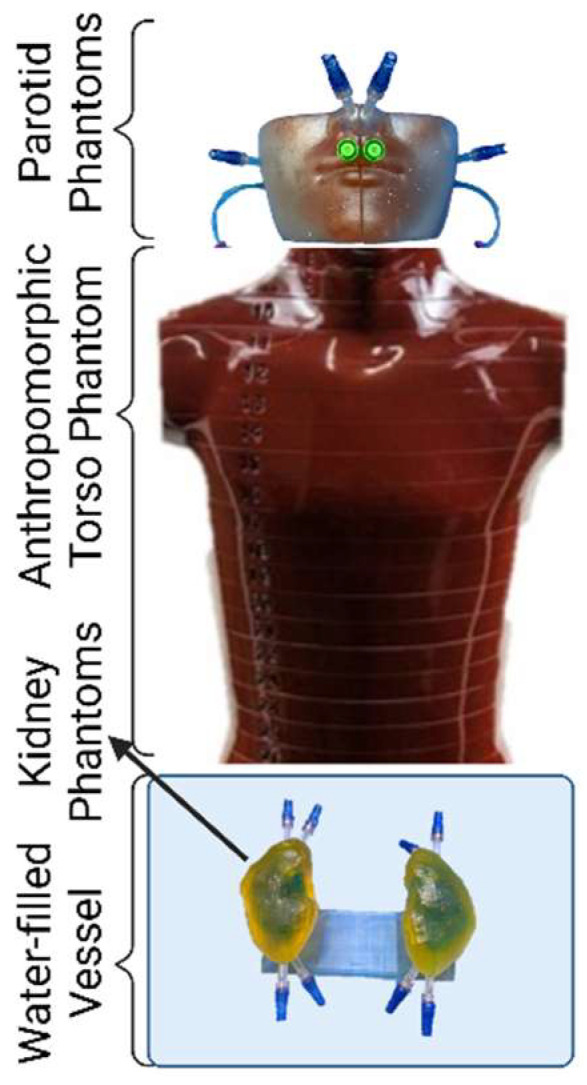
Schematic diagram of the phantom setup during image acquisition. Not to scale.

We acquired single-photon emission computed tomography (SPECT) and computed tomography (CT) images (referred to together as SPECT/CT) of the 3D-printed phantoms at 96 h post injection. We aimed to replicate full-scatter conditions during imaging by placing the kidney phantoms, supported by their base, in a vat of water abutting an anthropomorphic torso phantom (Alderson Radiation Therapy Male Phantom, Radiology Support Devices, Inc., Long Beach, CA, USA), which was adjacent to the parotid phantoms (Figure 6).

The SPECT/CT images were acquired on a Siemens Symbia Intevo Excel camera (Siemens Healthineers, Erlangen, Germany) with 3/8″ NaI crystal detectors fitted with a medium-energy low-penetration (MELP) parallel hole collimator. All acquisitions used the triple energy window (TEW) approach with a 20% window around the 208 keV photopeak, matching the highest-yield photon decay of ^177^Lu, and 10% scatter windows just above and below the photopeak window for scatter correction [36].

We acquired SPECT projections using a 180-degree non-circular (i.e., contoured) orbit, 60 views/head (3° per step), 20 s/view, 1.0× zoom, 128 × 128 matrix (4.795 mm isotropic voxel size). The transmission x-ray CT acquisition used a helical abdomen protocol at 130 kV with CARE Dose4D dose modulation with a helical pitch of 0.9. CT image reconstruction used filtered backprojection (FBP) with the Siemens B08s reconstruction convolution kernel, producing a CT slice thickness of 5 mm. Following image acquisition, we transferred NM projection images (all three energy windows) and the CT scan to MIM for quantitative reconstruction and processing.

#### 4.4.3. Quantitative Image Reconstruction

MIM SurePlan MRT with SPECTRA Quant software (Version 7.1.2) performed quantitative SPECT reconstruction of the TEW projection data. MIM SurePlan MRT is a commercially available software with FDA approval for quantitative SPECT image reconstruction and patient-specific dosimetry from 22 radionuclides, including ^177^Lu. SPECTRA Quant is the module within MIM SurePlan MRT that performs quantitative SPECT reconstruction. We previously calibrated SPECTRA Quant for SPECT/CT images of ^177^Lu-PSMA-617 collected with the scanner and acquisition settings used in this study following guidance from the developer (MIM Software Inc., Beachwood, OH, USA). The SPECT camera sensitivity calibration procedure comprises acquiring projections from a cylindrical phantom containing a known amount of radioactivity to identify the camera calibration factor in counts per second (cps) per unit radioactivity concentration (Bq/mL). Our calibration used a flanged Jaszczak cylindrical phantom (216 mm diam, 186 mm height, 6.7 L) with image-quality features removed. We filled the phantom with 2.5 uM aqueous ethylenediaminetetraacetic acid (EDTA) solution, to which we added 736.3 MBq of ^177^Lu and mixed well. Calibration image acquisition used a medium-energy collimator and triple energy window (TEW) acquisition protocol (20% window around photopeak, 10% upper/lower scatter windows). A 180-degree non-circular orbit collected 128 views/head (1.41 degrees/step), 10 s/view, 1.0× zoom, 128 × 128 matrix, for approximately 9 million total counts. The nuclear projection images for all three energy windows and CT of the Jaszczak phantom were provided to MIM for determination of the camera calibration factor.

SPECTRA Quant uses the determined calibration factor to execute an ordered subset expectation maximization (OSEM) reconstruction with six iterations, 20 subsets, and no 2D or 3D filters applied. The reconstruction incorporates energy window-based scatter correction, CT-based attenuation correction, and depth-dependent resolution recovery. The SPECTRA Quant reconstruction produces a quantitative image set with voxel values in units of Bq/mL. We used SPECTRA Quant to reconstruct the TEW NM projection images collected of our 3D-printed phantoms, in consideration of the associated CT image, into a quantitative image set for further analysis.

#### 4.4.4. Radioactivity Verification

We evaluated the accuracy of the SPECTRA Quant reconstruction for organ realistic geometries by comparing the radioactivity in the quantitative image set to the known injected radioactivity. We used MIM contouring tools to delineate the left and right whole kidneys, left and right kidney cortices, and the left and right parotids on the CT portion of the SPECT/CT image. We measured the radioactivity within each contour as the integral pixel magnitude of the quantitative image set. We decay-corrected the injected activities (Table 4) to the time of imaging and compared the imaged and decay-corrected injected activities using mean error and mean relative error. We calculated the recovery coefficient (*RC*) for each organ-realistic phantom as shown in Equation [1]:

(1) $$RC=\frac{A_{\mathrm{Imaged}}}{A_{\mathrm{Injected}}}$$

where $A_{\mathrm{Imaged}}$ was the radioactivity within the quantitative image and $A_{\mathrm{Injected}}$ was the known injected radioactivity, decay corrected to the time of imaging (Table 4). Finally, we considered the sensitivity of the *RC* to the injected radioactivity, chamber volume, and the ratio of the chamber surface area to chamber volume using standard least-squares linear regression analysis.

## 5. Conclusions

This study represents the first end-to-end test of a workflow for generating and implementing patient-derived fillable phantoms as a part of commissioning and quality assurance for patient-specific RPT dosimetry. Our findings demonstrated that 3D-printed organ-realistic phantoms are feasible and cost-effective. The results of this study can support other centers and clinics looking to implement or refine their patient-specific RPT dosimetry program by providing the tools needed to comprehensively evaluate accuracy in clinically relevant geometries. Looking forward, widespread accurate patient-specific RPT dosimetry programs will improve our understanding of RPT dose response and enable personalized RPT dosing to optimize patient outcomes.

## Figures and Tables

**Table 1:** Measured volumes of phantom chambers at three points in the phantom design and fabrication workflow. Note that the left and right kidney cortex models were generated in the computer-aided design (CAD) model and so volumes of original contours were unavailable. The right parotid was generated by mirroring the original left parotid contour. The CAD error was defined as the difference between the volume measured in the original patient contour and the volume of the CAD model, relative to the volume of the original patient contour such that positive numbers indicate an expansion from the original contour to the CAD model. The printing error was defined as the difference between the volume measured in the computed tomography (CT) image of the printed phantom and the volume of the CAD model, relative to the volume of the CAD model such that positive numbers indicate an expansion from the CAD model to the printed phantom.

Chamber	Original Contour (cc)	CAD Model (cc)	CAD Error (%)	CT of Printed Phantom (cc)	Printing Error (%)
Left Kidney Full	195.9	206.1	5.2	211.3	2.6
Right Kidney Full	192.4	197.7	2.8	200.9	1.6
Left Kidney Cortex	N/A	155.6	-	160.4	3.1
Right Kidney Cortex	N/A	149.0	-	151.3	1.5
Left Parotid	28.6	28.6	0	32.0	11.8
Right Parotid	28.6	28.6	0	30.4	6.1
Average Error			2.0		4.5

**Table 2:** Results of performance evaluation measurements. $A_{\mathrm{Injected}}$ represents the known injected radioactivity, decay corrected to the time of imaging. $A_{\mathrm{Imaged}}$ represents the integral pixel magnitude within each structure contour in the quantitative image. The Recovery Coefficient was calculated as in Equation (1).

Contour	$A_{\mathrm{Injected}}$ (MBq)	$A_{\mathrm{Imaged}}$ (MBq)	Recovery Coefficient
Left Kidney (whole)	167.92	155.72	0.93
Right Kidney (whole)	189.59	175.92	0.93
Left Kidney Cortex	167.92	142.81	0.85
Right Kidney Cortex	189.59	156.20	0.82
Left Parotid	57.13	48.76	0.85
Right Parotid	53.00	43.76	0.83

**Table 3:** Parameters used for three-dimensional printing using stereolithography.

Parameter	Value
Density of supports (unitless)	0.7
Touchpoint size (mm)	0.4
Internal Supports	Off
Layer thickness—Kidneys (µm)	100
Layer thickness—Parotids (µm)	50

**Table 4:** Radionuclide activities of ^177^Lu-PSMA-617 injected into each chamber of the 3D-printed phantoms. Activities were measured in a calibrated Capintec CRC-15R with a measurement precision of 0.21 MBq (previously determined via repeated measures). All other chambers were filled with water (i.e., radionuclide activity of 0 MBq).

Chamber	Radioactivity (MBq)
Left Kidney Cortex	253.82
Right Kidney Cortex	286.57
Left Parotid	86.36
Right Parotid	80.11

## Data Availability

The raw data supporting the conclusions of this article will be made available by the authors on request.
